# Associations of Dietary Patterns and Allergies With Asthma Among University Students in Bangladesh: A Cross‐Sectional Study

**DOI:** 10.1002/hsr2.72771

**Published:** 2026-07-04

**Authors:** Muslima Akter, Md. Muktarul, Abdullah Faruque

**Affiliations:** ^1^ Department of Pharmacy Jahangirnagar University Savar Bangladesh; ^2^ Department of Public Health and Informatics Jahangirnagar University Savar Bangladesh; ^3^ Centre for Medical Research & Development Dhaka Bangladesh

**Keywords:** allergic conditions, asthma, Bangladesh, dietary patterns, University students

## Abstract

**Background and Aims:**

Asthma is a major public health problem worldwide, with a growing burden among young adults in low‐ and middle‐income countries, including Bangladesh. Dietary patterns and allergic conditions may influence asthma‐related outcomes; however, evidence among university students is limited. This study aimed to determine the prevalence of asthma‐related respiratory symptoms and allergic conditions among Bangladeshi university students and to examine their associations with dietary intake and dietary patterns.

**Methods:**

A cross‐sectional study was conducted among 469 students at Jahangirnagar University, Savar, Dhaka, Bangladesh, using a non‐probability sampling approach across different faculties and academic years. Data was collected between March and June 2025 via a structured Bangla questionnaire based on the European Community Respiratory Health Survey and a food‐frequency questionnaire. An asthma symptom score (0–9) was derived from nine respiratory items. Multivariable logistic regression models were used to assess associations between dietary factors, allergic conditions, and asthma‐related outcomes after adjustment for sociodemographic and lifestyle variables. Dietary patterns were identified via principal component analysis.

**Results:**

Among the participants, 50.3% were female, and most were aged 21–25 years. Ever asthma was reported by 17.7% of the participants, and wheezing during the previous 12 months was reported by 16.4%. Dust allergies (54.5%) and food‐induced hypersensitivity (36.4%) were common. According to the adjusted analyses, seafood intake was associated with increasing odds of wheezing (aOR 3.04), whereas fruit intake (aOR 0.53) and butter consumption (aOR 0.46) were protective. Cold‐induced hypersensitivity, mustard oil intake and frequent antihistamine use were associated with nocturnal symptom. Fast food, meat, milk, almond oil intake, food allergies, and cold‐induced hypersensitivity were associated with antibiotic‐treated airway infections. Dietary pattern analysis revealed that a fruit–vegetable pattern was inversely associated with wheezing, whereas meat‐dominant and milk‐product patterns showed mixed associations with nocturnal symptoms and infections.

**Conclusion:**

Asthma‐related symptoms and allergic conditions are common among Bangladeshi university students. Dietary habits, cooking oils, and allergic conditions were significantly associated with respiratory outcomes. Many of these factors are potentially modifiable, highlighting opportunities for campus‐based prevention strategies and dietary interventions to reduce respiratory morbidity in young adults.

## 1 Introduction

Asthma, a chronic respiratory disease characterized by breathing difficulties, inflammation, and narrowing of bronchial passages, represents a significant global health burden, affecting millions of people worldwide [1, 2]. Its prevalence has been increasing, particularly in low‐ and middle‐income countries (LMICs), including Bangladesh, where its impact is disproportionately severe [3, 4]. The status of the respiratory system presents unique challenges for children. Its impact on their physical, social and emotional well‐being leads to diminished quality of life (QoL) and increased healthcare demands [5].

Globally, asthma affects a substantial proportion of young adults aged 15–39 years [6]. For example, a systematic analysis of the Global Burden of Disease Study 2019 highlighted the dynamic changes in asthma burden within this demographic [6]. In adults aged 18–45 years, the estimated global burden of asthma was approximately 4.3% from 2002 to 2003, signifying that around 4 out of every 100 persons aged 18 to 45 years are affected by asthma, with notable disability, health resource utilization, and diminished quality of life [7]. Given that young adults, particularly university students, are in a crucial period for establishing long‐term lifestyles and dietary patterns, understanding the factors influencing asthma in this group is imperative [8, 9].

Dietary patterns and allergic conditions are increasingly recognized as influential factors in asthma pathophysiology. The increasing prevalence of asthma and allergies, especially in Westernized societies and those transitioning to similar lifestyles, has led to hypotheses linking these increases to environmental and behavioral changes, including dietary modifications [10, 11]. Instead of focusing on individual foods or nutrients, which often yield conflicting evidence, dietary pattern analysis offers a more holistic approach by considering the synergistic effects of food combinations [11, 12, 13]. For example, studies have investigated the associations between “Western” dietary patterns (often characterized by high intake of processed foods, refined grains, and sugary drinks) and increased risk of asthma and allergic rhinitis [14, 15, 16]. Conversely, diets rich in fruits, vegetables, and healthy fats appear to have protective effects [2, 17, 18]. The pathophysiology of asthma involves a complex interplay of genetic, immunological, and environmental factors [1]. Various allergies (e.g., dust, food, cold, pet) are highly prevalent and can influence asthma risk and respiratory outcomes [19]. Therefore, investigating both dietary patterns and allergic conditions provides stronger and more comprehensive insights into asthma etiology and management [20].

In Bangladesh, university students are undergoing significant shifts in dietary habits due to urbanization and exposure to Westernized food cultures, coupled with challenges associated with hostel living [8, 9]. These changes include increased consumption of fast foods and reduced intake of fruits and vegetables, potentially impacting health outcomes [8]. Studies have documented dietary diversity and food consumption patterns among university students in Bangladesh, highlighting the need to address nutritional challenges in this population [8, 21]. The prevalence of asthma in the adult rural population of Bangladesh has remained at approximately 7% for over a decade, indicating that asthma is a persistent public health concern [22]. Despite the growing recognition of asthma as a major health issue, particularly among secondary school students [23, 24], there is a notable scarcity of context‐specific evidence from Bangladesh and the broader South Asian region, especially concerning university students and young adults who are underrepresented in asthma research [25]. Previous research on diet and asthma has shown mixed results, often due to variations in study populations and methodological approaches, including a focus on single nutrients versus overall dietary patterns [11, 12]. Therefore, this study aimed to examine the associations of dietary patterns and allergic conditions with asthma and asthma‐related respiratory outcomes among university students in Bangladesh.

## 2 Methods

### 2.1 Study Design, Population and Settings

This cross‐sectional study was conducted among 469 currently enrolled university students at Jahangirnagar University, Savar, Bangladesh, a large public residential institution located in a suburban/semi‐urban environment around 30 km away from the capital city. The university has an enrollment of more than 15,000 students with varied academic backgrounds. Participants were recruited using a non‐probability convenience sampling approach across different faculties and academic years. A non‐probability convenience sampling approach was employed due to the absence of a complete and accessible student registration list at the time of data collection, combined with time and resource constraints during the study period. Students were approached in classrooms, residential halls, cafeterias, and other common areas and were invited to participate voluntarily based on their availability during the data collection period. Before participation, the purpose of the study was explained and informed consent was obtained from all participants. While random selection from the whole student population was not conducted, attempts were made to enhance sample diversity by including students from different departments, academic years, residential halls, and both sexes. Data were collected between March 1, 2025, and June 30, 2025, using a semi‐structured self‐administered questionnaire translated into Bangla and distributed across all university faculties. Prior to the main data collection, the Bangla‐translated questionnaire was pilot tested among 30 university students who were not included in the final analysis, to assess clarity, comprehensibility, cultural appropriateness, and feasibility of administration. The pilot test confirmed the questionnaire was clear, feasible, and appropriate for the target population. Completed questionnaires were checked for completeness before inclusion in the final analysis. Cronbach's alpha was used to examine the internal consistency reliability of the questionnaire. The asthma‐related symptom items showed good reliability (Cronbach's α = 0.82), while the allergy‐related sensitivity items showed acceptable reliability (Cronbach's α = 0.77). The diet‐related items also demonstrated acceptable internal consistency reliability (Cronbach's α = 0.72). The questionnaire is provided as supporting material.

### 2.2 Sample Size Calculation

The sample size for this study was calculated via the single population proportion formula: *n* = (z^2^pq)/d^2^, where n is the required sample size, z is 1.96 for a 95% confidence interval, p is the expected prevalence, q = 1 − p, and d is the margin of error (0.05). Because robust prior prevalence data were limited, a conservative estimate of *p* = 0.50 was used to obtain the maximum sample size; therefore, q = 0.50. Substituting these values, *n* = [(1.96)^2^ × 0.50 × 0.50]/(0.05)^2^ = 384.16, which was rounded to 384. To account for a 10% nonresponse rate and possibly incomplete questionnaires, the adjusted sample size was calculated as 384 × 1.10 = 422.4, rounded to 423. In the present study, data were collected from 469 participants and included in the final analysis, indicating that the sample size achieved exceeded the minimum required sample size.

### 2.3 Assessment of Health Data

Similar self‐administered questionnaires have been used in previous studies conducted in Nigeria, Iran, and Bangladesh [26, 27, 28, 29]. The survey included questions from the European Community Respiratory Health Survey (ECRHS III), which asked about doctor‐diagnosed asthma, current asthma, allergens, respiratory symptoms, and other factors [30, 31]. According to the ECRHS III criteria, current asthma was defined as either the use of asthma medication or the experience of asthma attacks in the past 12 months [32]. Another set of questions asked about asthma‐related respiratory symptoms in the last year without mentioning asthma. The asthma‐related symptom items exhibited acceptable internal consistency reliability in the present study (Cronbach's α = 0.82). The responses were coded as “0” (no) or “1” (yes). Additionally, two questions addressed respiratory infections: the number of infections in the last 3 months and the number of infections requiring antibiotics in the past twelve months.

### 2.4 Asthma Symptom Score

An asthma symptom score (0–9) was created by summing the number of ‘yes’ responses to the following yes/no questions taken from the third European Community Respiratory Health Survey (ECRHS III) [33]:

1. Have you ever had asthma?
2. Have you experienced an asthma attack in the last 12 months?
3. Are you currently using asthma medications or inhaled steroids?
4. Have you experienced wheezing or whistling in your chest in the last 12 months?
5. Have you had a daytime attack of shortness of breath at rest in the last 12 months?
6. Have you had a daytime attack of shortness of breath after physical exercise in the last 12 months?
7. Have you woken up at night with a feeling of chest tightness in the last 12 months?
8. Have you woken up due to nocturnal attacks of shortness of breath in the last 12 months?
9. Have you woken up because of nocturnal attacks of cough in the last 12 months?

### 2.5 Assessment of Current Diet

The dietary questions used were taken from previous studies on the current diet [34]. This questionnaire includes 11 food frequency questions (FFQs) regarding the consumption of meat, fish, seafood, raw vegetables, cooked vegetables, milk, yogurt, fast food, fruit juice, and soft drinks. Each food item had five options: 0 = never, 1 = less than once a week, 2 = once a week, 3 = more than once a week, and 4 = daily. For presentation, the frequency responses were aggregated and recategorized into two groups: those that consumed ≥ 2 times per week and those that consumed < 2 times per week. In addition, the participants were asked five dichotomous (yes/no) questions regarding the types of cooking oils or fats they usually consume, including butter, olive oil, mustard oil, almond oil, and poly‐unsaturated oils.

### 2.6 Statistical Analysis

The associations between asthma symptoms, allergies, and dietary factors were analyzed via multivariable logistic and linear regression models, accounting for demographic, lifestyle, and family covariates. Initially, all FFQ scales, oil/fat questions, and allergy variables were included. Then, backward stepwise regression was used to retain only the significant or nearly significant predictors. For each one‐unit increase in scale score, odds ratios (ORs) with 95% confidence intervals (CIs) were calculated. For the FFQ, ORs were calculated for a one‐unit increase on a four‐point scale (0–4). The OR for allergy scales was calculated for a one‐unit increase on a 0–1 scale. The model's performance was evaluated via ROC analysis (AUC > 70%), and multicollinearity was checked (VIF < 5, with a range of 1.09–2.91). Kendall's tau beta was used to explore the relationships between patterns or FFQ measures. Dietary patterns were identified through factor analysis (principal component analysis with varimax rotation), with the number of components chosen on the basis of the scree plot and eigenvalues. The Kaiser–Meyer–Olkin (KMO) measure of sampling adequacy was 0.70, indicating acceptable suitability of the data for factor analysis. Factor loadings greater than 0.30 were considered meaningful for interpretation. All analyses were performed in R (v4.4.2) and were two‐tailed (α = 0.05).

### 2.7 Ethical Approval

Before the commencement of this study, ethical approval was obtained from the Biosafety, Biosecurity and Ethical Committee (BBEC), Faculty of Biological Sciences, Jahangirnagar University, Savar, Dhaka, Bangladesh (Reference No: BBEC, JU/M 2025/02 (181)).

## 3 Results

### 3.1 Sociodemographic Characteristics of the Study Participants

A total of 469 university students were included in the final analysis (Table 1). The majority of the participants were aged 21–25 years. Female students constituted 50.3% of the sample, whereas males accounted for 49.7%. Most participants belonged to middle‐income families (monthly income BDT 15,000–30,000). A large proportion of the students (84.0%) resided in university halls, and 63.1% reported rural residence during childhood. Smoking during the past month was reported by 7.7% of the participants, whereas 24.3% reported frequent use of antihistamine. A parental history of asthma or allergy was reported by 46.3% of the students.

**Table 1:** Distribution of students' socio‐demographic characteristics (*N* = 469).

Characteristics	Frequency (*n*)	Percentage (%)
Age		
Mean ± SD	22.7 ± 2.4	
15–20	94	20.1
21–25	307	65.6
> 25	68	14.3
Gender		
Male	233	49.7
Female	236	50.3
Monthly family income		
< 15000	109	23.3
15000–30000	185	39.4
> 30000	175	37.3
Faculty		
Arts	101	21.6
Science	361	76.9
Commerce	7	1.5
Current residential areas		
University hall	394	84.0
Hostel	16	3.4
At home	59	12.6
Childhood residential areas		
Village	298	63.5
City	171	36.5
Smoking status within the last month		
Yes	36	7.7
No	433	92.3
Antihistamine user		
Yes	114	24.3
No	355	75.7
Parental asthma/allergy		
Yes	217	46.3
No	252	53.7

### 3.2 Prevalence of Asthma‐Related Outcomes and Allergic Conditions

The distributions of asthma‐related outcomes and allergic conditions are presented in Table 2. Asthma was reported by 17.7% of the participants, whereas physician‐diagnosed asthma was reported by 13.2%. Asthma attacks during the previous 12 months were reported by 10.2% of the students, and current use of asthma medication was reported by 4.9%. Wheezing or whistling in the chest during the past 12 months was reported by 16.4% of the participants, and wheezing accompanied by breathlessness was reported by 13.8%. Daytime breathlessness was reported by 25.5% of the students. Nocturnal chest tightness, nocturnal breathlessness, and nocturnal cough were reported by 18.1%, 9.8%, and 32.3% of the participants, respectively.

**Table 2:** Prevalence of allergy, asthma and respiratory symptoms among Jahangirnagar University students (*n* = 469).

Variables	*n* (%)
Questions on asthma	
Ever had asthma	83 (17.7)
Ever had a doctor diagnose asthma	62 (13.2)
Attack of asthma last 12 months	48 (10.2)
Current asthma medicines or inhaled steroids	23 (4.9)
Respiratory symptoms (last 12 months)	
Wheeze or whistling in the chest	77 (16.4)
Wheeze combined with shortness of breath	65 (13.8)
Daytime attack of shortness of breath at rest	90 (19.1)
Daytime attack of shortness of breath after physical exercise	138 (29.4)
Any daytime breathlessness (rest or exercise)	120 (25.5)
Woken up at night with a feeling of chest tightness	85 (18.1)
Woken up by nocturnal attacks of shortness of breath	46 (9.8)
Woken up at night for coughing	152 (32.3)
Respiratory infections	
An airway infection lasted for 3 months	72 (15.3)
Airway infection with antibiotic treatment in the last 12 months	85 (18.1)
Any nasal allergies with constant sneezing	163 (34.7)
Food allergy	171 (36.4)
Cold‐induced hypersensitivity	160 (34.0)
Dust allergy	256 (54.5)
Furry pet allergy	106 (22.6)
Cat allergy	72 (15.3)
Dog allergy	77 (16.4)

Airway infection lasting at least 3 months was reported by 15.3% of the students, whereas airway infection requiring antibiotic treatment was reported by 18.1%. Allergic conditions, including dust allergies (54.5%), food allergies (36.4%), cold‐induced hypersensitivity (34.0%), nasal allergies with persistent sneezing (34.7%), furry pet allergies (22.6%), cat allergies (15.3%), and dog allergies (16.4%), were common among the participants.

### 3.3 Dietary Intake Patterns

The dietary intake patterns are summarized in Table 3. A total of 81.3% of the participants consumed fish at least twice per week, while 88.1% consumed cooked vegetables. Fruits and raw vegetables were consumed at least twice per week by 45.7% and 50.0% of the students, respectively. Fast food consumption was reported by 37.0% of the participants, and carbonated soft drink intake was reported by 33.6%. Milk and yogurt were consumed at least twice per week by 31.7% and 10.6% of the students, respectively.

**Table 3:** Frequency of dietary intake among university students (≥ 2 times/week).

Food item	*n* (%)
Meat	358 (76.4)
Fish	381 (81.3)
Seafood	35 (7.4)
Fruits	214 (45.7)
Raw vegetables	234 (50.0)
Cooked vegetables	413 (88.1)
Milk	149 (31.7)
Yogurt	50 (10.6)
Fast food	174 (37.0)
Fruit juice	79 (16.8)
Carbonated soft drinks	158 (33.6)

### 3.4 Associations Between Dietary Intake, Allergies, and Asthma‐Related Outcomes

Multivariable logistic regression analysis was performed to evaluate associations of asthma‐related outcomes with dietary habits and allergic conditions, which are presented in Supplementary Table S1. Several dietary and allergy variables were substantially linked with respiratory symptoms. Wheeze was positively associated with seafood intake (aOR 3.15, 95% CI; 1.04–8.92, *p* = 0.03) and daytime breathlessness was inversely associated with fruit intake (aOR 0.50, 95% CI; 0.29–0.87, *p* = 0.01). Consumption of raw vegetables was inversely associated with awakening with chest tightness at night (aOR: 0.49, 95% CI; 0.27‐0.87, *p* = 0.01). Consumption of mustard oil was positively linked with nocturnal waking with chest tightness (aOR: 2.33, 95% CI; 1.27 to 4.42, *p* = 0.007) and nocturnal cough attacks (aOR: 2.70, 95% CI; 1.64 to 4.56, *p* < 0.001). Polyunsaturated oil intake was inversely associated with nocturnal cough attacks (aOR: 0.38, 95% CI; 0.17–0.87, *p* = 0.02). Food allergy was associated with daytime dyspnoea (aOR: 1.93, 95% CI; 1.12 to 3.32, *p* = 0.01). Cold‐induced hypersensitivity was positively associated with wheeze (aOR: 3.05, 95% CI; 1.48–6.48, *p* = 0.003), daytime breathlessness (aOR: 3.98, 95% CI; 2.19–7.41, *p* < 0.001), waking at night with chest tightness (aOR: 2.09, 95% CI; 1.05–4.20, *p* = 0.03), nocturnal attacks of cough (aOR: 2.11, 95% CI; 1.21–3.72, *p* = 0.008) and airway infection requiring antibiotic treatment (aOR: 2.60, 95% CI; 1.37–5.04, *p* = 0.003). Recent exposure to smoking in the past month was associated with daytime breathlessness (aOR: 3.13, 95% CI; 1.16–9.99, *p* = 0.03).

### 3.5 Multivariable Logistic Regression Analysis

The multivariate backward stepwise logistic regression results are presented in Table 4. Seafood consumption was positively associated with odds of wheezing (aOR 3.04; *p* = 0.02). Fruit (aOR 0.53; *p* = 0.02) and butter consumption (aOR 0.46; *p* = 0.02) were inversely associated with odds of wheezing. Cat allergies (aOR 2.45; *p* = 0.008), cold‐induced hypersensitivity (aOR 2.61; *p* = 0.002), antihistamine use (aOR 2.44; *p* = 0.003), and smoking during the past month (aOR 3.83; *p* = 0.01) were positively associated with increased odds of wheezing.

**Table 4:** Multivariable backward stepwise logistic regression showing factors associated with asthma‐related outcomes.

Exposure variable	Adjusted OR (95% CI)	*p* value
Wheeze		
Seafood	3.04 (1.12–7.48)	0.02
Fruits	0.53 (0.29–0.92)	0.02
Butter	0.46 (0.23–0.87)	0.02
Cold‐induced hypersensitivity	2.61 (1.42–4.81)	0.002
Cat allergy	2.45 (1.25–4.74)	0.008
Antihistamine use	2.44 (1.34–4.46)	0.003
Smoking in last one month	3.83 (1.43–13.48)	0.01
Any daytime breathlessness (rest or exercise)		
Fruits	0.60 (0.37–0.97)	0.03
Food allergy	1.83 (1.11–3.00)	0.01
Cold‐induced hypersensitivity	3.87 (2.25–6.78)	< 0.001
Smoking in last one month	3.80 (1.77–9.19)	0.01
Nocturnal chest tightness		
Raw vegetables	0.52 (0.30–0.86)	0.01
Mustard oil	1.99 (1.13–3.64)	0.02
Cold‐induced hypersensitivity	1.88 (1.05–3.36)	0.03
Cat allergy	2.43 (1.28–4.56)	0.005
Antihistamine use	2.18 (1.22–3.87)	0.007
Childhood residence (village)	0.53 (0.31–0.92)	0.02
Nocturnal cough		
Butter	0.56 (0.34–0.90)	0.02
Mustard oil	2.37 (1.49–3.88)	< 0.001
Poly‐unsaturated oils	0.41 (0.19–0.87)	0.01
Cold‐induced hypersensitivity	2.09 (1.28–3.41)	0.002
Antihistamine use	1.74 (1.05–2.87)	0.02
Smoke	5.15 (1.79–18.96)	0.005
Childhood residence (village)	0.58 (0.37–0.92)	0.02
Airway infection requiring antibiotics		
Meat	2.00 (1.03–4.16)	0.04
Milk	1.88 (1.08–3.23)	0.02
Fast food	1.82 (1.02–3.25)	0.04
Almond oil	2.71 (1.29–5.56)	0.006
Food allergy	1.84 (1.04–3.26)	0.03
Cold‐induced hypersensitivity	2.47 (1.36–4.53)	0.003
Antihistamine use	1.92 (1.06–3.48)	0.02

*Note:* All models were adjusted for age, sex, parental asthma/allergy, childhood and current residential areas, smoking status and family structure. Variables shown were retained in the final model after backward stepwise selection. CI: confidence interval; OR: odds ratio.

For daytime breathlessness, fruit intake (aOR 0.60; *p* = 0.03) was inversely associated, whereas food allergy (aOR 1.83; *p* = 0.01), cold‐induced hypersensitivity (aOR 3.87; *p* < 0.001) and smoking during the past month (aOR 3.80; *p* = 0.01) increased the odds ratio.

Nocturnal chest tightness was inversely associated with raw vegetable intake (aOR 0.52; *p* = 0.01) and childhood village residence (aOR 0.53; *p* = 0.02). Mustard oil intake (aOR 1.99; *p* = 0.02), cat allergy (aOR 2.43; *p* = 0.005), cold‐induced hypersensitivity (aOR 1.88; *p* = 0.03) and antihistamine use (aOR 2.18; *p* = 0.007) were associated with increased odds.

For nocturnal cough, butter (aOR 0.56; *p* = 0.02), poly‐unsaturated oils (aOR 0.41; *p* = 0.01), and childhood village residence (aOR 0.58; *p* = 0.02) were inversely associated, whereas mustard oil intake (aOR 2.37; *p* < 0.001), cold‐induced hypersensitivity (aOR 2.09; *p* = 0.002), frequent antihistamine use (aOR 1.74; *p* = 0.02), and ever‐smoker (aOR 5.15; *p* = 0.005) were positively associated.

Airway infection requiring antibiotic treatment was positively associated with meat intake (aOR 2.00; *p* = 0.04), milk intake (aOR 1.88; *p* = 0.02), fast food consumption (aOR 1.82; *p* = 0.04), almond oil intake (aOR 2.71; *p* = 0.006), food allergy (aOR 1.84; *p* = 0.03), cold‐induced hypersensitivity (aOR 2.47; *p* = 0.003), and antihistamine use (aOR 1.92; *p* = 0.02).

### 3.6 Associations Between Dietary Patterns and Asthma‐Related Outcomes

The dietary pattern–based associations are presented in Table 5. Dietary patterns were derived using principal component analysis, and the factor loading matrix used to label the extracted dietary patterns is presented in Supplementary Table S3. The fruit–vegetable dietary pattern was inversely associated with wheeze (aOR 0.69; 95% CI: 0.50–0.96; *p* = 0.02) and wheeze with breathlessness (aOR 0.64; 95% CI: 0.44–0.93; *p* = 0.02). The milk products dietary pattern was positively associated with nocturnal breathlessness (aOR 1.66; 95% CI: 1.10–2.51; *p* = 0.01). In addition, the meat consumption dietary pattern was positively associated with airway infection requiring antibiotic treatment (aOR 1.45; 95% CI: 1.10–1.96; *p* = 0.01). Additional associations with allergic outcomes are presented in Supplementary Table S2.

**Table 5:** Associations between dietary patterns and asthma‐related outcomes based on factor analysis.

Dietary pattern (factor)	Adjusted OR (95% CI)	*p* value
Wheeze		
Factor 3: Fruit–vegetable dietary pattern	0.69 (0.50–0.96)	0.02
Antihistamine use	4.55 (2.69–7.75)	0.001
Wheeze with breathlessness		
Factor 3: Fruit–vegetable dietary pattern	0.64 (0.44–0.93)	0.02
Antihistamine use	8.58 (4.79–15.74)	< 0.001
Nocturnal breathlessness		
Factor 6: Milk products dietary pattern	1.66 (1.10–2.51)	0.01
Antihistamine use	6.99 (3.62–13.93)	< 0.001
Nocturnal cough		
Factor 1: fast food & quick sugar	1.27 (0.98–1.66)	0.06
Parental asthma/allergy	1.59 (1.05–2.42)	0.02
Childhood in the village	0.65 (0.42–0.99)	0.04
Antihistamine use	2.22 (1.40–3.50)	< 0.001
An airway infection that required antibiotic treatment		
Factor 2: butter & oils	0.76 (0.58–1.03)	0.07
Factor 5: meat consumption	1.45 (1.10–1.96)	0.01

*Note:* All models were adjusted for age, sex, parental asthma/allergy, childhood and current residential areas, and family structure. Variables shown were retained in the final model after backward stepwise selection. Dietary patterns were derived using principal component analysis. CI: confidence interval; OR: odds ratio.

## 4 Discussion

A key finding from this study was the positive association between seafood consumption and increased odds of wheezing. This observation diverges from those of some studies, particularly those from Western countries, which often highlight the protective role of certain seafoods due to their omega‐3 fatty acid content, which is known for its anti‐inflammatory properties [2]. However, the relationship between seafood and asthma is complex. In some contexts, specific seafoods can be common allergens, and preparation methods can influence their allergenic potential [2]. For example, a European study examining dietary patterns and respiratory health did not find a consistent association between fish consumption and asthma outcomes across all populations, suggesting regional variations in dietary composition and potential allergenicity [11]. To investigate whether food allergies influenced this association, additional analyses showed that the connection between seafood consumption and wheeze remained unchanged when food allergy was excluded from the multivariable model (0% change in the seafood coefficient), indicating that food allergy was unlikely to be a confounder of this association. The specific types of seafood consumed and cooking practices used in Bangladesh may have contributed to these divergent findings.

Conversely, fruit intake consistently demonstrated an inverse association with both wheezing and daytime breathlessness. Similarly, raw vegetable intake was inversely associated with nocturnal chest tightness. These findings are highly consistent with extensive international literature emphasizing the anti‐inflammatory and antioxidant benefits of diets rich in fruits and vegetables in improving respiratory health [2, 10]. For example, the GA^2^LEN study, involving nine European countries, identified a beneficial effect of fruit and vegetable intake on asthma and allergic symptoms [11]. In a Mexican cohort, adherence to a Mediterranean dietary pattern characterized by high fruit and vegetable consumption was associated with reduced asthma and rhinitis in children [18]. Given the shifting dietary habits among university students in Bangladesh, promoting increased fruit and raw vegetable consumption could be a vital public health strategy [8, 9].

Allergic conditions have emerged as critical determinants of asthma‐related outcomes. Cat allergy was significantly associated with increased odds of wheezing and nocturnal chest tightness. This finding is highly consistent with the well‐established role of pet dander as a potent allergen in triggering allergic asthma globally [1]. Food allergies are associated with increased odds of daytime breathlessness and airway infection requiring antibiotic treatment. This highlights that food allergies can not only directly trigger respiratory symptoms but also potentially increase susceptibility to more severe airway infections, possibly through chronic inflammation or immune dysregulation [1]. The high prevalence of allergic conditions such as dust allergies (54.5%), food allergies (36.4%), and cold‐induced hypersensitivity (34.0%) among the Bangladeshi university students in our study underscores their significant public health relevance in this population. Our finding that cold‐induced hypersensitivity consistently serves as a risk factor across various outcomes, such as wheezing, daytime breathlessness, nocturnal cough, and antibiotic‐treated airway infections, is consistent with findings from Bangladesh, which highlighted the high prevalence of allergic disorders and possible environmental triggers [19]. These findings suggest that cold sensitivity, which is often linked to nonallergic airway hyperresponsiveness, is a substantial contributor to asthma symptom burden in this population. The current investigation found a strong correlation between smoking status and the prevalence of asthma symptoms. This finding aligns with other findings indicating that smoking during adolescence and adulthood increases the likelihood of developing asthma and exacerbates this condition [35].

Examining dietary patterns provides a broader perspective beyond individual food items. The fruit–vegetable dietary pattern was inversely associated with wheezing and breathlessness, further reinforcing the protective role of healthy eating habits against asthma symptoms. This aligns with a meta‐analysis identifying healthy dietary patterns rich in fruits and vegetables as protective against asthma risk in European populations [12, 13]. Similarly, a study in Singapore/Malaysia reported that a frequent plant‐based food intake pattern reduced the risk for atopic dermatitis exacerbation, indicating broader allergic benefits [17]. However, a surprising positive association was observed between the fruit–vegetable pattern and airway infection requiring antibiotics. This unexpected finding necessitates further research to explore potential reverse causation, confounding by other factors, or specific components within this pattern that might influence infection susceptibility. The milk product dietary pattern showed complex associations and was positively associated with nocturnal breathlessness but inversely associated with nocturnal cough. These nuanced and sometimes conflicting associations highlight the complexity of dietary pattern analysis and their potential interactions with immunological and environmental factors. For example, increased individual milk intake and fast‐food consumption is also associated with airway infections requiring antibiotic treatment. This suggests that while generally healthy patterns such as those rich in fruits and vegetables are beneficial, specific dietary elements or patterns, particularly those associated with more Westernized diets (e.g., fast food), may contribute to adverse respiratory outcomes, including increased susceptibility to airway infections. This trend is consistent with findings from Qatar, where fast food and sweet intake was directly associated with asthma incidence [15], and with studies from Iran, which linked a Western dietary pattern to allergic rhinitis in young women and asthma in children [14, 16]. These observations are particularly relevant for countries such as Bangladesh that are undergoing rapid dietary transitions [8, 21].

## 5 Limitations

Our study, like many others, has several limitations. Because of its cross‐sectional design, causal relationships between dietary factors, allergic conditions, and asthma outcomes could not be established. The findings were based on self‐reported symptoms and exposures, which may have introduced recall bias and misclassification. Another limitation is, a non‐probability convenience sampling technique was used, which may affect the generalisability of the findings and selection bias. Participants were selected on the basis of accessibility and desire to engage rather than by random selection. Objective clinical assessments such as spirometry and allergy testing were not performed because of logistical and funding constraints. In addition, the study was conducted at a single university, which may limit generalizability. Nevertheless, the results provide useful preliminary insight into asthma‐related patterns and modifiable risk factors among university students.

## 6 Conclusion

This study determined the prevalence of asthma‐related symptoms among Bangladeshi university students and evaluated associated dietary and allergic factors. A considerable proportion of the students reported respiratory symptoms and allergic conditions. Sociodemographic, lifestyle, dietary, and allergy‐related factors were significantly associated with asthma outcomes. Many of these determinants are potentially modifiable, and targeted improvements in diet, allergy awareness, and health behaviors may reduce the burden of respiratory morbidity. The findings provide important insight into student health and can inform campus‐based prevention strategies and public health planning aimed at lowering disease‐related impacts and healthcare costs.

## Author Contributions

**Muslima Akter:** conceptualization, data curation, investigation, formal analysis, writing – original draft. **Md. Muktarul:** conceptualization, methodology, investigation, formal analysis, writing – original draft, writing – review and editing, supervision. **Abdullah Faruque:** conceptualization, writing – review and editing, supervision.

## Funding

The authors have nothing to report.

## Ethics Statement

Ethical approval for this study was obtained from the Biosafety, Biosecurity and Ethical Committee (BBEC), Faculty of Biological Sciences, Jahangirnagar University, Savar, Dhaka, Bangladesh.

## Conflicts of Interest

The authors declare no conflicts of interest.

## Author Declaration

All authors have read and approved the final version of the manuscript. Md. Muktarul had full access to all the data in the study and takes complete responsibility for the integrity of the data and the accuracy of the data analysis.

## Artificial Intelligence Disclosure

An artificial intelligence–based language model (ChatGPT, OpenAI) was used to assist with language editing and clarity of presentation. The authors reviewed and take full responsibility for the content.

## Transparency Statement

**Md. Muktarul** affirms that this manuscript is an honest, accurate, and transparent account of the study being reported; that no important aspects of the study have been omitted; and that any discrepancies from the study as planned (and, if relevant, registered) have been explained.

## Supporting information


Supporting File 1

Supporting File 2

Supporting File 3

## Data Availability

The data that support the findings of this study are available from the corresponding author upon reasonable request. The data supporting the findings of this study are available from the corresponding author upon reasonable request. The study questionnaire and related materials are provided as Supporting Information. Any additional analytic code used in this study is available from the corresponding author upon reasonable request.
