# Supplementary material for: Associations of Dietary Patterns and Allergies With Asthma Among University Students in Bangladesh: A Cross‐Sectional Study
Source: Health Sci Rep. 2026 Jul 4;9(7):e72771. doi: 10.1002/hsr2.72771 (PMC13332859; doi:10.1002/hsr2.72771)
Supplement: Supplementary file 1 — Supporting File 1 [file HSR2-9-e72771-s001.docx]

**Supplementary Table S1. Adjusted associations between dietary intake, allergies, and asthma-related outcomes among university students**

| **Variables** | **Wheeze OR (95% CI); p value** | **Any daytime breathlessness**  **(rest or exercise)**  **OR (95% CI);**  **p value** | **Woken up at night with a feeling of chest tightness**  **OR (95% CI);**  **p value** | **Woken up by nocturnal attacks of cough**  **OR (95% CI);**  **p value** | **An airway infection that required antibiotic treatment**  **OR (95% CI);**  **p value** |
| --- | --- | --- | --- | --- | --- |
| Meat | 1.35 (0.67-2.83); 0.40 | 0.98 (0.56-1.75); 0.95 | 0.65 (0.35-1.22); 0.17 | 1.35 (0.79-2.34); 0.27 | 2.05 (1.01-4.39); 0.05 |
| Fish | 0.80 (0.39-1.71); 0.56 | 0.95 (0.51-1.78); 0.87 | 0.91 (0.47-1.87); 0.80 | 0.63 (0.36-1.14); 0.12 | 1.45 (0.69-3.18); 0.33 |
| Seafood | 3.15 (1.04-8.92); 0.03 | 1.02 (0.35-2.71); 0.96 | 2.08 (0.74-5.46); 0.14 | 0.97 (0.37-2.44); 0.95 | 1.25 (0.41-3.51); 0.67 |
| Fruits | 0.60 (0.31-1.17); 0.13 | 0.50 (0.29-0.87); 0.01 | 0.65 (0.35-1.18); 0.16 | 0.82 (0.49-1.35); 0.44 | 1.12 (0.61-2.09); 0.70 |
| Raw vegetables | 0.79 (0.42-1.46); 0.44 | 0.55 (0.33-0.91); 0.02 | 0.49 (0.27-0.87); 0.01 | 0.78 (0.48-1.25); 0.30 | 0.86 (0.47-1.56); 0.61 |
| Cooked vegetables | 0.68 (0.29-1.67); 0.38 | 1.36 (0.63-3.09); 0.44 | 1.23 (0.53-3.10); 0.64 | 2.02 (0.94-4.58); 0.07 | 1.19 (0.45-3.45); 0.73 |
| Milk | 0.88 (0.42-1.77); 0.72 | 1.63 (0.91-2.93); 0.09 | 1.02 (0.53-1.93); 0.96 | 1.09 (0.64-1.87); 0.73 | 1.61 (0.85-3.09); 0.14 |
| Yoghurt | 1.60 (0.53-4.56); 0.38 | 0.64 (0.23-1.64); 0.36 | 2.03 (0.75-5.22); 0.15 | 0.54 (0.21-1.34); 0.19 | 2.02 (0.75-5.28); 0.15 |
| Fast foods | 0.52 (0.26-1.03); 0.06 | 0.91 (0.54-1.55); 0.74 | 0.70 (0.38-1.27); 0.25 | 1.31 (0.79-2.14); 0.28 | 1.93 (1.05-3.56); 0.03 |
| Fruit juices | 0.74 (0.28-1.83); 0.53 | 1.62 (0.75-3.41); 0.20 | 0.89 (0.39-1.99); 0.78 | 1.28 (0.63-2.58); 0.48 | 0.69 (0.27-1.59); 0.39 |
| Carbonated soft drinks | 1.35 (0.66-2.70); 0.39 | 0.89 (0.49-1.57); 0.69 | 1.70 (0.91-3.19); 0.09 | 1.37 (0.80-2.34); 0.24 | 0.55 (0.27-1.11); 0.10 |
| Butter | 0.39 (0.18-0.82); 0.01 | 0.73 (0.41-1.26); 0.26 | 0.64 (0.33-1.21); 0.18 | 0.51 (0.29-0.88); 0.01 | 0.65 (0.32-1.25); 0.20 |
| Olive oil | 1.07 (0.29-3.58); 0.91 | 0.78 (0.26-2.22); 0.65 | 0.52 (0.14-1.66); 0.29 | 1.90 (0.71-5.12); 0.19 | 1.23 (0.39-3.76); 0.71 |
| Mustard oil | 1.76 (0.93-3.42); 0.08 | 1.33 (0.80-2.25); 0.26 | 2.33 (1.27-4.42); 0.007 | 2.70(1.64-4.56); <0.001 | 1.25 (0.68-2.34); 0.47 |
| Almond oil | 1.19 (0.41-3.16); 0.74 | 1.30 (0.55-2.97); 0.53 | 0.98 (0.35-2.48); 0.98 | 0.69 (0.29-1.54); 0.37 | 2.83 (1.18-6.69); 0.01 |
| Poly-unsaturated oils | 0.68 (0.24-2.12); 0.47 | 0.73 (0.31-1.80); 0.47 | 0.81 (0.31-2.44); 0.69 | 0.38 (0.17-0.87); 0.02 | 0.83 (0.30-2.60); 0.73 |
| Food allergy | 1.61 (0.83-3.07); 0.15 | 1.93 (1.12-3.32); 0.01 | 1.00 (0.54-1.87); 0.97 | 1.07 (0.64-1.77); 0.78 | 1.90 (1.03-3.52); 0.03 |
| Cold-induced hypersensitivity | 3.05 (1.48-6.48); 0.003 | 3.98 (2.19-7.41); <0.001 | 2.09 (1.05-4.201); 0.03 | 2.11 (1.21-3.723); 0.008 | 2.60 (1.37-5.04); 0.003 |
| Dust allergy | 0.89 (0.41-1.92); 0.78 | 0.56 (0.30-1.03); 0.06 | 1.22 (0.62-2.41); 0.56 | 1.21 (0.71-2.07); 0.47 | 1.75 (0.88-3.55); 0.11 |
| Furry pet allergy | 1.57 (0.59-3.96); 0.34 | 1.60 (0.72-3.51); 0.23 | 1.24 (0.50-2.92); 0.63 | 1.29 (0.58-2.78); 0.52 | 1.86 (0.75-4.54); 0.17 |
| Cat allergy | 2.12 (0.72-6.38); 0.17 | 1.12 (0.41-3.00); 0.82 | 2.09 (0.74-5.98); 0.16 | 1.28 (0.48-3.39); 0.61 | 0.52 (0.17-1.54); 0.24 |
| Dog allergy | 0.816 (0.30-2.08); 0.67 | 1.04 (0.61-1.75); 0.51 | 0.99 (0.39-2.38); 0.98 | 0.44 (0.18-1.02); 0.06 | 0.99 (0.38-2.54); 0.99 |
| Parental asthma/ allergy | 0.68 (0.36-1.29); 0.24 | 1.04 (0.61-1.75); 0.88 | 0.72 (0.39-1.29); 0.26 | 1.48 (0.91-2.41); 0.11 | 0.94 (0.50-1.72); 0.82 |
| Smoking last one month | 4.05 (1.02-27.42); 0.08 | 3.13 (1.16-9.99); 0.03 | 1.38 (0.49-4.61); 0.56 | 1.68 (0.71-4.34); 0.25 | 1.14 (0.24-5.65); 0.86 |
