# Supplementary material for: Associations of Dietary Patterns and Allergies With Asthma Among University Students in Bangladesh: A Cross‐Sectional Study
Source: Health Sci Rep. 2026 Jul 4;9(7):e72771. doi: 10.1002/hsr2.72771 (PMC13332859; doi:10.1002/hsr2.72771)
Supplement: Supplementary file 2 — Supporting File 2 [file HSR2-9-e72771-s002.docx]

# Supplementary Table S2. Additional associations between dietary patterns and allergic outcomes

| **Dietary pattern / factor** | **Adjusted OR (95% CI)** | **p value** |
| --- | --- | --- |
| **Furry pet allergy** |  |  |
| Factor 2: Butter & oil dietary pattern | 0.69 (0.53–0.91) | 0.009 |
| Factor 6: Milk product dietary pattern | 1.61 (1.19–2.17) | 0.001 |
| **Cat allergy** |  |  |
| Factor 4: Fish dietary pattern | 0.70 (0.54–0.91) | 0.007 |
| Factor 6: Milk product dietary pattern | 1.45 (1.03–2.03) | 0.03 |

All models were adjusted for age, sex, parental asthma/allergy, childhood and current residential areas, and family structure. Variables shown were retained in the final model after backward stepwise selection.
