# Supplementary material for: Associations of Dietary Patterns and Allergies With Asthma Among University Students in Bangladesh: A Cross‐Sectional Study
Source: Health Sci Rep. 2026 Jul 4;9(7):e72771. doi: 10.1002/hsr2.72771 (PMC13332859; doi:10.1002/hsr2.72771)
Supplement: Supplementary file 3 — Supporting File 3 [file HSR2-9-e72771-s003.docx]

**Supplementary table S3: Associations between the factors and the dietary components**

|  | F1 (fast food and quick sugar) | F2 (butter and oils) | F3 (vegetables and fruits | F4 (fish consumption) | F5 (meat consumption) | F6 (milk products) |
| --- | --- | --- | --- | --- | --- | --- |
| Meat |  |  |  |  | 0.982 |  |
| Fish |  |  |  | 0.994 |  |  |
| Seafood |  |  |  |  |  |  |
| Fruits |  |  | 0.728 |  |  |  |
| Raw vegetables |  |  | 0.448 |  |  |  |
| Cooked vegetables |  |  | 0.387 |  |  |  |
| Milk |  |  |  |  |  | 0.430 |
| Yoghurt |  |  |  |  |  | 0.710 |
| Fast foods | 0.542 |  |  |  |  |  |
| Fruit juices | 0.519 |  |  |  |  |  |
| Carbonated soft drinks | 0.681 |  |  |  |  |  |
| Butter |  | 0.421 |  |  |  |  |
| Olive oil |  | 0.705 |  |  |  |  |
| Mustard oil |  |  |  |  |  |  |
| Almond oil |  | 0.552 |  |  |  |  |
| Poly-unsaturated oils |  |  |  |  |  |  |
